# p53 Positivity Predicts Poor Survival in Oropharyngeal Squamous Cell Carcinoma Dependent on HPV Status

**DOI:** 10.3390/cancers18101660

**Published:** 2026-05-20

**Authors:** Lilianny Querino Rocha de Oliveira, Fatemeh Farshadi, Alex Mlynarek, Marco A. Mascarella, Michael Hier, Ricardo D. Coletta, Sabrina Daniela Silva Wurzba

**Affiliations:** 1 Department of Otolaryngology Head and Neck Surgery, Sir Mortimer B. Davis-Jewish General Hospital, McGill University, Montreal, QC H3T 1E2, Canada (L.Q.R.d.O.); sabrina.wurzba@mcgill.ca (S.D.S.W); 2 Lady Davis Institute for Medical Research, Sir Mortimer B. Davis-Jewish General Hospital, McGill University, Montreal, QC H3T 1E2, Canada; 3 Graduate Program in Oral Biology, School of Dentistry, University of Campinas, Piracicaba 13414-903, SP, Brazil; 4 Division of Experimental Medicine, Department of Medicine and Health Sciences, McGill University, Montreal, QC H4A 3J1, Canada; 5 Department of Oral Diagnosis, School of Dentistry, University of Campinas, Piracicaba 13414-903, SP, Brazil

**Keywords:** oropharyngeal cancer, human papilloma virus, p16, p53, outcomes

## Abstract

**Background:** Human papillomavirus (HPV)-associated oropharyngeal squamous cell carcinoma (OPSCC) generally has a more favorable prognosis compared to HPV-negative cases. Overexpression of p16^INK4a^ is widely used as a surrogate marker for HPV-induced carcinogenesis, and it also represents an important tumor suppressor gene, the second most frequently altered after *TP53*. In turn, mutations in *TP53* are characteristic of tumors linked to smoking and alcohol consumption and p53 expression is consistently associated with worse clinical outcomes. **Aims:** Investigate the impact of p53 immunohistochemical expression on prognosis of OPSCC. **Methods:** This retrospective study included 155 OPSCC patients with longitudinal follow-up exceeding 10 years. Immunohistochemistry was used to evaluate p53 protein expression. Clinicopathological associations were performed to evaluate the prognostic impact of p53 protein expression in OPSCC HPV-positive and HPV-negative tumors. **Results:** Among the 155 OPSCC cases, 90 (58.1%) were classified as HPV-positive and 65 (41.9%) as HPV-negative. HPV status was inversely associated with p53 positivity, with HPV-negative tumors showing a higher frequency of p53 expression (*p* < 0.0001). Patients with HPV-positive tumors experienced better clinical outcomes than those with HPV-negative disease, including cancer-specific survival (CSS; HR: 3.47, 95% CI 1.16–10.4, *p* = 0.02) and disease-free survival (DFS; HR: 3.73, 95% CI 1.29–10.7, *p* = 0.01), whereas p53 positivity alone was not independently associated with survival. Notably, individuals with HPV-negative and p53-positive tumors exhibited the poorest outcomes, in contrast to patients with HPV-positive OPSCC, regardless of p53 expression. **Conclusions:** Patients with HPV-negative and p53-positive OPSCC showed inferior clinical outcomes compared with their HPV-positive counterparts, independent of p53 status. These findings underscore the prognostic relevance of jointly evaluating HPV status and p53 expression in OPSCC and support more refined risk stratification and personalized therapeutic strategies.

## Simple Summary

Oropharyngeal cancer related to human papillomavirus generally has a better prognosis than cancers caused by smoking and alcohol consumption. However, not all patients experience favorable outcomes, and additional markers are needed to improve risk prediction. In this study, we evaluated the expression of the p53 protein together with human papillomavirus status in 155 patients with oropharyngeal cancer followed for more than 10 years. We found that patients with tumors negative for human papillomavirus and positive for p53 had the worst clinical outcomes, including lower survival and higher recurrence rates. In contrast, p53 expression alone was not sufficient to predict prognosis. Our findings suggest that evaluating human papillomavirus and p53 together may improve patient stratification and help identify individuals who could benefit from closer monitoring and more personalized treatment strategies.

## 1. Introduction

Head and neck cancer (HNC) develops in various anatomical sites within the upper aerodigestive tract, such as the oral cavity, larynx, pharynx, and nasal/paranasal sinuses, and represents around 5% of all malignancies in the world [1,2]. Several risk factors contribute to HNC, including tobacco and alcohol abuse, viral infections, such as Epstein–Barr virus and human papillomavirus (HPV), betel nut chewing, immunodeficiency, radiation exposure, diet, and genetic predisposition [3,4]. These factors are directly associated with the molecular pathogenesis of HNC, including in cases linked to high-risk HPV infection, such as oropharyngeal squamous cell carcinomas (OPSCC) [5,6].

**Figure 1:**
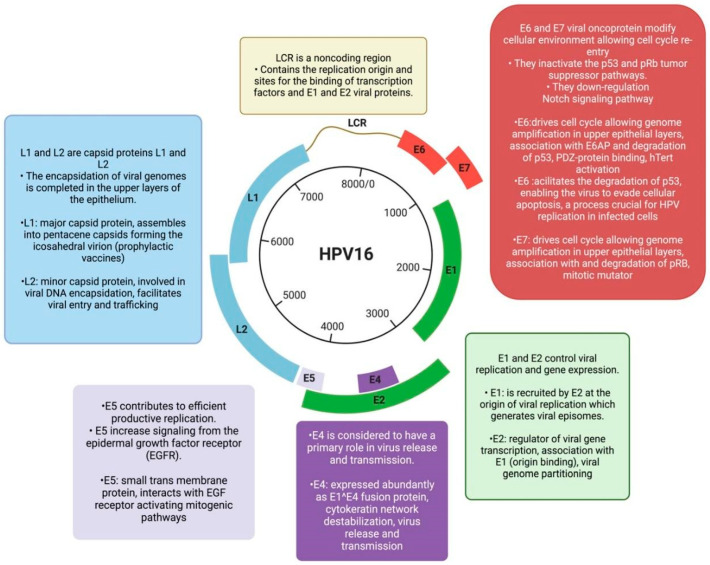
Schematic representation of the circular genome organization of the Human Papillomavirus type 16 (HPV16). The Long Control Region (LCR) is a noncoding region that contains the origin of replication and binding sites for transcription factors and viral proteins E1 and E2. E1 and E2 oncoproteins are crucial for viral replication and gene expression. E1 binds to the origin of replication to generate viral episomes, while E2 regulates viral transcription and assists in viral genome division in coordination with E1. The E4 protein, expressed as an E1–E4 fusion protein, plays a key role in viral release by destabilizing the cytokeratin network. E5, a small transmembrane protein, enhances replication by activating mitogenic pathways through Epidermal Growth Factor Receptor (EGFR) signaling. The E6 and E7 oncoproteins modify the cellular environment, promoting cell cycle re-entry by inactivating tumor suppressor proteins p53 and Rb and downregulating Notch signaling. E6 degrades p53 to evade apoptosis and facilitate genome amplification, while E7 promotes cell cycle progression and genome amplification through Rb degradation. The L1 and L2 capsid proteins form the viral capsid, with L1 being the major structural protein, used in prophylactic vaccines, and L2 aiding in viral DNA encapsulation, entry, and trafficking. Created with BioRender (biorender.com).

HPV, especially HPV-16, is recognized as a significant driver of oncogenesis in OPSCC, contributing to distinct molecular pathways that differ from those of tobacco- and alcohol-induced OPSCC [7,8]. HPV is characterized as a circular double-stranded DNA virus with an 8 kb genome that encodes several viral proteins, including two oncoproteins (E6 and E7) as well as two capsid proteins (L1 and L2) [9,10,11]. The viral genome is encapsulated within an icosahedral structure approximately 55 nm in diameter, formed by L1 and L2 proteins [12,13]. These capsid proteins facilitate the interaction between the virus and host cells [14,15,16]. When the genetic material of HPV integrates into the host cell’s DNA, the viral oncoprotein E7 binds to and inactivates the retinoblastoma protein (Rb) [17,18,19]. This disruption leads to cell cycle arrest and consequently the overexpression of the tumor suppressor protein p16, which serves as a surrogate marker for HPV-positive OPSCC [20,21,22]. Although p16 immunohistochemistry is widely used in clinical practice and incorporated into the current AJCC staging system, more specific methods, such as HPV in situ hybridization (HPV-ISH), are also available for HPV detection [23,24,25]. In addition to E7, the viral oncoprotein E6 further contributes to cell cycle dysregulation by promoting the degradation of the p53 protein [26,27,28] (Figure 1).

**Figure 2:**
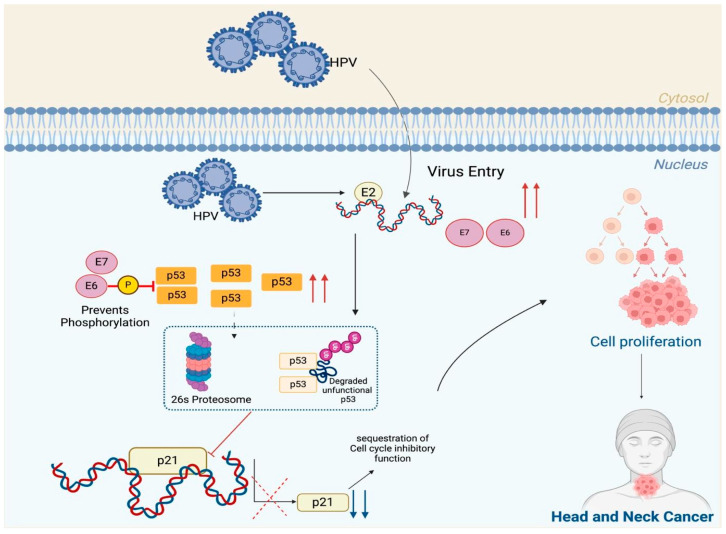
Schematic illustration of the mechanism by which Human Papillomavirus (HPV) contributes to the development of head and neck cancer (HNC). Upon HPV entry into the host cell, the viral DNA is released and undergoes transcription, leading to the production of viral oncoproteins E6 and E7. These oncoproteins disrupt key cellular regulatory pathways. E6 binds to and promotes the degradation of the tumor suppressor protein p53 through the 26S proteasome, inhibiting its phosphorylation and reducing functional p53 levels. The loss of p53 function impairs the activation of p21, a cyclin-dependent kinase inhibitor, which normally regulates the cell cycle. The combined effects of E6 and E7 impair normal cell cycle control, resulting in increased cell proliferation and contributing to the development of head and neck cancer. Created with BioRender (biorender.com).

p53, a key transcription factor, plays a critical role in tumor suppression by regulating the expression of target genes involved in DNA repair and cell cycle control [29,30,31,32]. In HPV-negative OPSCC, 65–85% of *TP53* mutations are missense mutations, which result in the substitution of a single amino acid [33,34,35]. These mutations not only disrupt the tumor-suppressive function of wild-type protein but also confer new oncogenic properties, facilitating tumor recurrence and chemoresistance [36] (Figure 2). HPV-positive OPSCC often retain a functional level of wild-type p53, which could contribute to their increased radiosensitivity and potentially lead to improved treatment outcomes [37,38,39]. The interplay between p16 and p53 provides critical insights into the molecular heterogeneity of OPSCC. While p16 overexpression is commonly associated with better outcomes in HPV-positive cases, the prognostic role of p53 expression remains inconsistent across cohorts and requires further validation, particularly in studies with long-term follow-up [40,41]. Evidence suggests that the co-expression of p16 and p53 could characterize a subset of OPSCC with more aggressive behavior [24,25]; however, it remains unclear whether this interaction improves or reduces the prognostic value of p16 as a standalone marker.

Previous studies evaluating the prognostic significance of p53 in OPSCC, particularly in combination with HPV or p16 status, have reported conflicting results [19,42,43]. While HPV-positive tumors are consistently associated with improved clinical outcomes [19], the independent role of p53 expression remains controversial. Some studies suggest that p53 overexpression is associated with poorer prognosis, especially in HPV-negative tumors [42], whereas others have reported no significant association or even improved survival in specific subsets of OPSCC [19,43]. These discrepancies may be explained by differences in cohort composition, methods used to assess p53 status, and the biological distinction between HPV-driven and non-HPV-driven tumors.

Moreover, the combined evaluation of p16 and p53 has been proposed as a more reliable approach for distinguishing HPV-related OPSCC, as HPV-positive tumors typically show p16 overexpression with reduced or absent p53 expression [19,42]. In this context, using multiple biomarkers rather than isolated markers may enhance diagnostic classification and improve prognostic stratification in head and neck cancers. Building on this framework, the present study evaluates how HPV status and p53 expression jointly relate to clinical outcomes in OPSCC. Specifically, the findings of this study suggest that p53 positivity identifies a subgroup of HPV-negative OPSCC patients with poorer prognosis.

## 2. Materials and Methods

### 2.1. Patient Samples

Tumor samples were collected from 155 patients with OPSCC, diagnosed and treated at the Jewish General Hospital, Faculty of Medicine (McGill University) in Montreal, Quebec, Canada, between 2009 and 2014 (more than 10 years of follow-up). The study was conducted following the Declaration of Helsinki; Scientific Research Ethics Committees of the Centre intégré universitaire de santé et de services sociaux du Centre-Ouest-de-l’Île-de-Montréal (MEO-37-2022-2938) reviewed and approved this study. Eligibility criteria included previously untreated OPSCC patients submitted for treatment in the same institution without any distant metastasis at diagnosis (M0). The tumor staging was re-classified according to the International Union Against Cancer (TNM) and grouped as early clinical stage (I + II) or advanced clinical stage (III + IV). Patients treated with chemoradiotherapy or radiotherapy received standard institutional protocols. Radiation doses typically ranged from 66 to 70 Gy to the primary tumor region, while elective neck regions received 50–56 Gy. Concurrent radiosensitizing chemotherapy consisted mainly of cisplatin-based regimens. Surgical management varied according to tumor location and disease stage and included procedures such as TORS (transoral robotic surgery), including radical tonsillectomy and oropharyngectomy, neck dissection and search for unknown primary. The medical records were the main source to obtain detailed clinicopathological information. Strengthening the reporting of observational studies (STROBE Statement) was used to ensure appropriate methodological quality (http://www.strobe-statement.org/).

### 2.2. Tissue Microarray (TMA) Construction

TMAs were constructed by extracting 2.0 mm cores from microscopically defined representative areas of OPSCC from formalin-fixed paraffin-embedded (FFPE) and corresponding morphologically normal epithelium from tumor-free adjacent margins. Using the Tissue Microarrayer^®^ (Beecher Instruments, Silver Springs, MD, USA), duplicate cores were punched and arrayed onto a single recipient paraffin block, with each core spaced 0.2 mm apart. After sectioning the recipient block, the slides were coated with a layer of paraffin to prevent oxidation and stored at −20 °C.

### 2.3. Immunohistochemistry

Immunohistochemical staining was performed on the TMA slides as previously described [44]. Briefly, the slides were incubated overnight at 4 °C with primary antibodies diluted in PBS: anti-p16 (E6H4, CINTEC p16 Histology Kit, Roche, Switzerland), and anti-p53 (Bp53-11 PaB, Roche/Ventana Medical Systems, Tucson, AZ, USA). After incubation, secondary antibodies (AdvancedTMHRP Link, DakoCytomation, Carpinteria, CA, USA) were applied for 30 min, followed by the polymer detection system (AdvancedTMHRP Link, DakoCytomation) for another 30 min at room temperature. The reactions were visualized using a DAB solution (0.6 mg/mL, Sigma, St. Louis, MO, USA) with 0.01% H_2_O_2_ and counterstained with hematoxylin. Positive controls were included for all reactions following the manufacturer’s guidelines, and negative controls were established by omitting the primary antibody and substituting it with normal serum.

The reactions were replicated on separate TMA slides to capture different tissue levels of the same lesion, with the second slide being 25–30 sections deeper, providing a 300 μm distance between the two, allowing for a 4-fold redundancy. Tissue cores were examined at ×100 magnification (×10 objective and ×10 eyepiece). HPV status was determined based on p16 expression. p53 expression was considered positive when strong nuclear staining was observed, whereas p16 expression was considered positive when strong and diffuse nuclear and cytoplasmic staining was present in tumor cells [23]. Immunohistochemical quantification and stratification were additionally performed using digital H-score analysis based on staining intensity and percentage of positive cells.

### 2.4. QuPath Analysis

TMA slides were scanned to generate high-resolution digital images and analyzed using QuPath software (v0.2.2). The TMA dearrayer function was initially applied to identify the grid layout, followed by manual exclusion of damaged cores and staining artifacts. Tumor cells were manually annotated and separated from non-tumor cells to ensure that only tumor cells were included in the analysis. The positive cell detection algorithm was then used to quantify positive and negative cells in each sample. Cell staining intensity was classified based on predefined thresholds of DAB optical density (OD) mean. The proportion of positive cells was calculated by dividing the number of positive cells by the total number of cells. Tumors were considered p53-negative when <5% of tumor cells showed p53 positivity, and p53-positive when ≥5% of tumor cells were positive.

### 2.5. Statistical Analysis

Associations between p53 positivity and clinicopathological tumor characteristics were assessed using cross-tabulation analyses and the chi-square test, as appropriate. Overall survival (OS), cancer-specific survival (CSS), and disease-free survival (DFS) were estimated using the Kaplan–Meier method and compared between groups using the log-rank test. Survival analyses were performed using Cox proportional hazards regression models. Initially, univariate analyses were conducted to evaluate the association between each clinicopathological variable and survival outcomes. Variables considered clinically relevant or associated with survival in univariate analyses were subsequently included in the multivariable Cox regression models. A stepwise selection approach was applied to identify the final multivariable model. Variables incorporated into the analyses included HPV status, p53 expression, age, sex, smoking exposure, alcohol consumption, tumor stage, nodal status, and treatment-related variables. Hazard ratios (HRs) and corresponding 95% confidence intervals (95% CIs) were calculated. To further explore the prognostic interaction between HPV status and p53 expression, subgroup analyses combining both biomarkers were also performed. Statistical significance was defined as a two-sided *p*-value ≤ 0.05 throughout all analyses.

## 3. Results

### 3.1. Patient Characteristics

**Figure 3:**
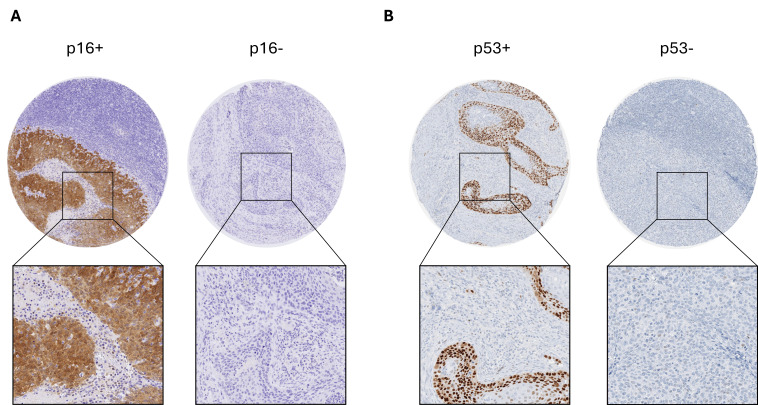
Representative immunohistochemical staining for p16 and p53. The top panels show the full TMA cores, and the boxed regions indicate a magnified 100 µm area. (**A**) The left panel shows p16-positive staining, while the right panel shows p16-negative staining. (**B**) The left panel shows p53-positive staining, and the right panel shows p53-negative staining. The cases shown in (**A**,**B**) correspond to the same tissue sample.

The demographic and clinicopathologic features of the patients included in this study are described in Table 1. The study included 155 patients, with a mean age of 60.8 years (standard deviation of 10.6), ranging from 34 to 87 years. Males represent 74.8% (*n* = 116) of the sample, and the tonsil was the most common tumor subsite, accounting for 91 cases (58.7%). At the time of the initial diagnosis, 131 patients (84.5%) were diagnosed with advanced-stage disease (stage III and IV). The histopathological tumor grading classified 35 (22.6%) as well-differentiated, 71 (45.8%) as moderately differentiated, and 49 (31.6%) as poorly differentiated. Immunohistochemical p16-positivity was found in 90 (58.1%) tumors, and 65 (41.9%) were negative for p16. Representative samples classified as p16-negative and p16-positive are depicted in Figure 3. Tobacco use was reported by 105 (67.7%) patients, and alcohol consumption was confirmed by 96 (61.9%). The mean follow-up period was 153.3 months, ranging from 1 to 211 months, during which 38 patients (24.5%) died, 17 of causes unrelated to OPSCC. Recurrence (any type) was identified in 21 (13.5%) patients.

### 3.2. Association of p53 Positivity with OPSCC Clinicopathological Features

The p53 immunohistochemical expression was localized exclusively in the nucleus of the tumor cells, with variable distribution and intensity (Figure 3). Regarding this immunoreactivity, 80 patients (51.6%) were classified as negative and 75 (48.4%) as positive. Positive p53 expression was significantly associated with sex, being observed in 66.7% of male cases compared with 33.3% of female cases (*p* = 0.02). p53 positivity was also strongly associated with HPV status (*p* < 0.0001) (Table 2). Specifically, p53 positivity was approximately 1.8 times more frequent in HPV-negative than in HPV-positive OSCC. No significant associations were observed between p53 positivity and age, smoking or drinking habits, tumor site, treatment modality, histological grade, survival status, or recurrence (Table 2).

### 3.3. Survival Analysis

**Figure 4:**
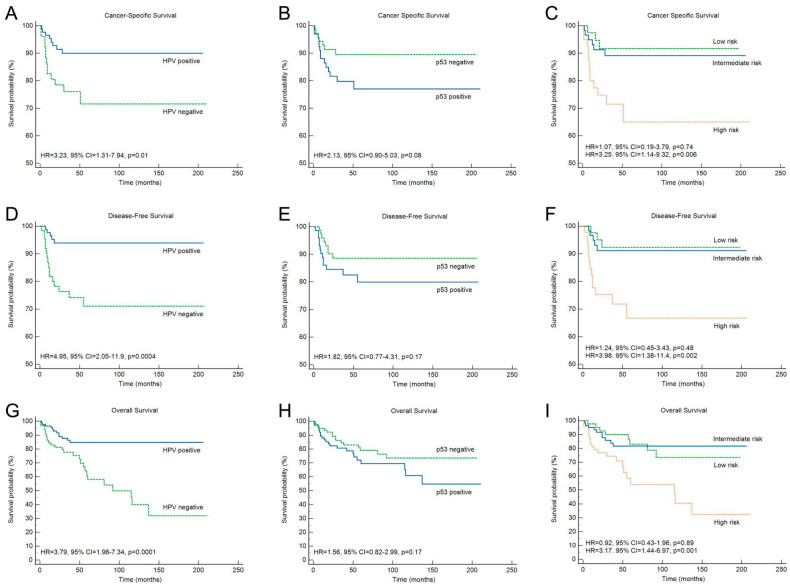
Survival analyses in oropharyngeal cancer patients. Kaplan–Meier curves show that HPV-positive tumors are associated with better cancer-specific survival (**A**,**B**), disease-free survival (**D**,**E**), and overall survival (**G**,**H**) compared with HPV-negative tumors. (**C**,**F**,**I**) Combined HPV/p53 risk classification stratified patients into low risk (HPV-positive/p53-negative), intermediate risk (HPV-positive/p53-positive or HPV-negative/p53-negative), and high risk (HPV-negative/p53-positive). High-risk patients showed significantly poorer CSS, DFS, and OS in both univariate and multivariate analyses, highlighting the prognostic value of integrating HPV status with p53 expression.

Univariate survival analysis for CSS demonstrated a significant association between poorer prognosis and several factors, including age (HR = 3.38, 95% CI 1.24–9.25, *p* = 0.02), smoking (HR = 5.85, 95% CI 1.36–25.1, *p* = 0.02), tumors located at the base of the tongue (HR = 4.68, 95% CI 1.81–12.1, *p* = 0.0007), treatment with chemotherapy alone (HR = 16.1, 95% CI 4.43–58.7, *p* < 0.0001), and HPV status (HR = 2.98, 95% CI 1.24–7.22, *p* = 0.01) (Table 3). Similar results were observed for OS, with the addition of a significant association with histopathological tumor grading. In univariate analysis, smoking (HR = 10.9, 95% CI 1.46–81.3, *p* = 0.02), tumor location at the base of the tongue (HR = 3.74, 95% CI 1.42–9.84, *p* = 0.007) or soft palate (HR = 3.48, 95% CI 1.55–7.78, *p* = 0.002), and HPV status (HR = 5.12, 95% CI 1.87–13.9, *p* = 0.002) were predictive of poorer DFS (Table 3). Kaplan–Meier analyses demonstrated that HPV-positive tumors were associated with significantly better CSS, DFS, and OS compared with HPV-negative tumors (Figure 4).

The multivariate survival analysis based on the stepwise model of the Cox regression test revealed that smoking (HR = 4.65, 95% CI 1.07–20.1, *p* = 0.04), location of the tumor (HR = 2.30, 95% CI 1.10–4.78, *p* = 0.03) and HPV status (HR = 3.47, 95% CI 1.16–10.4, *p* = 0.02) were independent prognostic factors for CSS (model 1, Table 4). Smoking habit, tumor location and HPV status were also independently associated with DFS, whereas only tumor location remained associated with OS (model 1, Table 4).

To investigate the prognostic value of HPV status in combination with p53 expression, we conducted both univariate and multivariate survival analyses. Tumors were classified into three risk groups based on this combination: low risk (HPV-positive/p53-negative), intermediate risk (either HPV-positive/p53-positive or HPV-negative/p53-negative), and high risk (HPV-negative/p53-positive). In univariate analysis, patients in the high-risk group exhibited significantly poorer outcomes compared with the low-risk group, including CSS (HR = 1.81, 95% CI 1.11–2.97, *p* = 0.01), DFS (HR = 2.01, 95% CI 1.19–3.36, *p* = 0.008), and OS (HR = 1.75, 95% CI 1.20–2.57, *p* = 0.004) (Table 3). Multivariate analysis confirmed that the combination of HPV status and p53 positivity was independently associated with CSS, DFS, and OS (model 2, Table 4). Kaplan–Meier survival curves further demonstrated the discriminative power of this combined classification in stratifying patient outcomes (Figure 4).

## 4. Discussion

HPV-positive OPSCC presents distinct clinical features compared to HPV-negative cases. It primarily affects younger individuals who typically have no history of smoking or alcohol use, and tends to respond more favorably to chemotherapy and radiation therapy [38]. As a result, patients with HPV-positive OPSCC often experience improved survival rates and better overall outcomes [39,40]. These differences are often attributed to the preservation of wild-type p53 function, lower mutational burden, and distinct molecular profiles relative to carcinogen-driven tumors, particularly those associated with tobacco and alcohol exposure [45]. Evaluating HPV status is, thereafter, considered essential in OPSCC management [23,41], with immunohistochemical detection of p16 widely recognized as a reliable surrogate marker for HPV-driven oncogenesis [24]. Given this strong correlation, all analyses in this study were stratified by HPV (p16 expression) status of the patients.

The primary objective of this study was to investigate the prognostic impact of p53 immunohistochemical expression in OPSCC, considering its interaction with HPV status. Our findings revealed that p53 expression alone did not demonstrate a prognostic value, an observation consistent with studies reporting heterogeneous results when p53 is evaluated without considering the tumor’s etiological context [24,25]. However, when p53 expression was assessed in conjunction with HPV status, a strong prognostic pattern emerged: patients with HPV-negative/p53-positive tumors showed the poorest outcomes, including reduced CSS, DFS, and OS. Conversely, p53 positivity did not negatively impact outcomes in HPV-positive patients, reinforcing the notion that p53 immunostaining in this context carries a distinct biological interpretation. In HPV-driven tumors, the viral E6 protein promotes degradation of wild-type p53, and immunohistochemical detection of p53 may instead reflect transient stabilization or cellular stress responses rather than true TP53 mutation [46,47]. Therefore, interpretation of p53 immunostaining should always take HPV status into account to avoid misclassification when analyzed in isolation. The integration of HPV and p53, in a model, demonstrated strong discriminatory capacity across all survival endpoints, outperforming the prognostic value of either biomarker alone. In addition to its prognostic implications, the combined assessment of p16 and p53 may improve the interpretation of immunohistochemical results. Pakkanen et al. [48] demonstrated that simultaneous evaluation of these markers allows accurate subclassification of head and neck squamous cell carcinomas into HPV-associated and HPV-independent tumors, as p53 staining patterns are heterogeneous and reflect underlying molecular alterations. When interpreted alongside p16 status, these patterns may increase diagnostic confidence and improve tumor classification.

Smoking emerged as an important risk modifier, being strongly associated with increased specific mortality, higher recurrence rate, and a significant reduction in overall survival in our model. These findings are aligned with the meta-analysis by Chen et al. [49], which also identified worse overall survival, worse disease-free survival, and worse specific survival among smokers with HPV-positive OPSCC, in addition to a trend towards increased locoregional recurrence. Similarly, when considering only HPV-positive populations, Anantharaman et al. [50] estimated that the difference in OPSCC prevalence between non-smokers and smokers is 0.5% among HPV E6-negative individuals and 8.8% among HPV E6-positive individuals, reinforcing the role of smoking as a relevant prognostic factor regardless of viral status. This stratified approach aligns with contemporary efforts in head and neck oncology to refine prognostic prediction through combined biomarker signatures rather than single-marker evaluation [23,46]. The adverse prognostic effect observed in this study was predominantly confined to the HPV-negative/p53-positive subgroup, rather than to p53 positivity across the overall OPSCC cohort. These findings suggest that the prognostic relevance of p53 immunohistochemistry is highly context-dependent and may be enhanced when interpreted in conjunction with HPV status. Nevertheless, despite its potential utility for risk stratification, the evidence remains insufficient to support the routine implementation of p53 assessment as a universal prognostic biomarker across all HNSCC cases. Notably, the clear identification of a high-risk subgroup within the HPV-negative population represents a relevant contribution, given the limited availability of robust prognostic markers for these patients. As observed in other populations [51], the current study observed that OPSCC was more frequent in men, with the highest prevalence occurring between the sixth and seventh decades of life. We also found a predominance of HPV-positive cases compared to HPV-negative ones, reflecting the growing incidence of this subtype in Western countries, driven by the increasing burden of HPV-related oropharyngeal infections [52,53]. Furthermore, tonsillar tumors represented the predominant anatomical subsite among HPV-positive OPSCC cases, corroborating prior evidence supporting the strong association between HPV infection and the tonsillar crypt epithelium [47].

This study has several important strengths, including a well-characterized cohort with long-term follow-up exceeding 10 years, rigorous pathological review, and the use of redundant TMA sampling to enhance the robustness and reproducibility of biomarker assessment. Nevertheless, certain limitations should be acknowledged. Although the findings provide clinically relevant insights, validation in larger prospective and multicenter cohorts remains necessary before these biomarkers can be incorporated into routine clinical decision-making or formal risk stratification algorithms. In addition, p53 immunohistochemistry alone cannot discriminate between missense and truncating TP53 alterations, nor distinguish true TP53 mutations from wild-type p53 stabilization. Therefore, complementary molecular characterization, including TP53 sequencing, would be valuable to more precisely define the biological and clinical significance of the high-risk subgroup identified in this study.

Despite these limitations, our results support the concept that the prognostic value of p53 is context-dependent and substantially strengthened when interpreted alongside HPV status. In particular, the identification of the HPV-negative/p53-positive subgroup as having markedly poorer outcomes highlights a potentially clinically relevant population that may benefit from closer surveillance and future risk-adapted therapeutic approaches. Collectively, these findings contribute to the growing body of evidence supporting integrated molecular stratification as a framework for advancing precision oncology in OPSCC. This approach aligns with emerging risk-adapted treatment paradigms, in which biomarker-defined low-risk patients may be considered for de-escalation while higher-risk groups could warrant treatment intensification.

## 5. Conclusions

In summary, the findings of this study demonstrated that HPV-negative and p53-positive OPSCCs exhibited worse outcomes, including shortened CSS and higher recurrence risk, compared with HPV-positive tumors, regardless of their p53 expression. These results underscore the complexity of HPV-associated OPSCC, where molecular markers such as p53 provide additional prognostic insights beyond HPV status alone. Our findings emphasize the importance of incorporating HPV and p53 stratification into prognosis and treatment planning. By doing so, personalized therapeutic strategies can be optimized, improving outcomes and tailoring interventions to the unique profile of HPV-associated OPSCC.

## Figures and Tables

**Table 1:** Clinicopathological features of the sample with 155 oropharyngeal squamous cell carcinomas.

	*n*	%
Age (years)		
Mean ± SD	60.8 ± 10.6	
Range	34–87	
Sex		
Male	116	74.8
Female	39	25.2
Smoking		
No	50	67.7
Yes	105	32.3
Alcohol		
No	59	38.1
Yes	96	61.9
Clinical stage		
Early (I + II)	14	9.0
Advanced (III + IV)	131	84.5
Missing data	10	6.5
Location		
Tonsil	91	58.7
Base of tongue	60	38.7
Soft palate	4	2.6
Treatment		
Chemotherapy + Radiotherapy	111	71.6
Chemotherapy	3	1.9
Surgery	6	3.9
Radiotherapy	18	11.6
Surgery + Radiotherapy	10	6.5
Surgery + Radiotherapy + Chemotherapy	7	4.5
Histopathological grading		
Well-differentiated	35	22.6
Moderately differentiated	71	45.8
Poorly differentiated	49	31.6
HPV status		
Positive	90	58.1
Negative	65	41.9
Recurrence		
No	134	86.5
Yes	21	13.5
Status		
Alive	117	75.5
Cancer-specific death	21	13.5
Non-cancer death	17	11.0

**Table 2:** Association of clinicopathological features of oropharyngeal squamous cell carcinoma with positivity of p53.

	p53 Negative *n* (%)	p53 Positive *n* (%)	*p*-Value
Age			
<61 years	41 (51.3)	35 (46.7)	
≥61 years	39 (48.7)	40 (53.3)	0.57
Sex			
Male	66 (82.5)	50 (66.7)	
Female	14 (17.5)	25 (33.3)	0.02
Smoking			
No	27 (33.7)	23 (30.7)	
Yes	53 (66.3)	52 (69.3)	0.68
Alcohol consumption			
No	34 (42.5)	25 (33.3)	
Yes	46 (57.5)	50 (66.7)	0.24
Clinical stage			
Early (I + II)	9 (11.7)	5 (7.4)	
Advanced (III + IV)	68 (88.3)	63 (92.6)	0.38
Location			
Tonsil	49 (61.2)	42 (56.0)	
Base of tongue	30 (37.5)	30 (40.0)	
Soft palate	1 (1.3)	3 (4.0)	0.50
Treatment			
Chemotherapy + Radiotherapy	55 (68.8)	56 (74.8)	
Chemotherapy	2 (2.5)	1 (1.3)	
Surgery	2 (2.5)	4 (5.3)	
Radiotherapy	12 (15.0)	6 (8.0)	
Surgery + Radiotherapy	6 (7.5)	4 (5.3)	
Surgery + Radiotherapy + Chemotherapy	3 (3.7)	4 (5.3)	0.64
Histopathological grading			
Well-differentiated	15 (18.8%)	20 (26.7)	
Moderately differentiated	39 (48.7%)	32 (42.7)	
Poorly differentiated	26 (32.5%)	23 (30.6)	0.48
HPV status			
Positive	63 (78.7)	27 (36.0)	
Negative	17 (21.3)	48 (64.0)	<0.0001
Recurrence			
No	72 (90.0)	62 (82.7)	
Yes	8 (10.0)	13 (17.3)	0.18
Status			
Alive	64 (80.0)	53 (70.7)	
Cancer-specific death	7 (8.8)	14 (18.7)	
Non-cancer death	9 (11.2)	8 (10.6)	0.19

**Table 3:** Univariate analysis for cancer-specific survival, disease-free survival and overall survival of patients with oropharyngeal squamous cell carcinoma.

	Cancer-Specific Survival		Disease-Free Survival		Overall Survival	
	HR (95% CI)	*p*-Value	HR (95% CI)	*p*-Value	HR (95% CI)	*p*-Value
Age						
<61 years	1		1		1	
≥61 years	3.38 (1.24–9.25)	0.02	1.14 (0.48–2.69)	0.75	2.26 (1.15–4.44)	0.02
Sex						
Male	1		1		1	
Female	0.67 (0.27–1.65)	0.38	0.69 (0.28–1.72)	0.44	0.69 (0.35–1.37)	0.29
Smoking						
No	1		1		1	
Yes	5.85 (1.36–25.1)	0.02	10.9 (1.46–81.3)	0.02	6.89 (2.12–22.4)	0.001
Alcohol consumption						
No	1		1		1	
Yes	1.69 (0.65–4.36)	0.28	2.29 (0.83–6.26)	0.11	1.44 (0.74–2.82)	0.29
Clinical stage						
Early (I + II)	1		1		1	
Advanced (III + IV)	3.04 (0.02–19.1)	0.96	1.04 (0.24–4.48)	0.96	1.61 (0.38–6.77)	0.52
Location						
Tonsil	1		1		1	
Base of tongue	4.68 (1.81–12.1)	0.0007	3.74 (1.42–9.84)	0.007	3.36 (1.71–6.62)	0.0005
Soft palate	NA	0.99	3.48 (1.55–7.78)	0.002	1.39 (0.50–3.87)	0.52
Histopathological grading						
Well-differentiated	1		1		1	
Moderately differentiated	0.92 (0.29–2.88)	0.88	0.54 (0.19–1.49)	0.24	0.44 (0.22–0.91)	0.03
Poorly differentiated	0.82 (0.43–1.56)	0.55	0.77 (0.44–1.33)	0.36	0.64 (0.42–0.98)	0.04
Treatment						
Chemotherapy + Radiotherapy	1		1		1	
Chemotherapy	16.1 (4.43–58.7)	<0.0001	5.74 (0.75–44.1)	0.09	13.9 (3.99–48.8)	<0.0001
Surgery	1.41 (0.51–3.01)	0.51	1.47 (0.71–3.08)	0.29	1.55 (0.85–2.82)	0.15
Radiotherapy	1.00 (0.61–1.65)	0.98	0.72 (0.37–1.41)	0.34	1.09 (0.79–1.51)	0.58
Surgery + Radiotherapy	1.22 (0.84–1.77)	0.31	0.96 (0.57–1.59)	0.87	1.20 (0.88–1.62)	0.24
Surgery + Radiotherapy + Chemotherapy	0.09 (0.01–314.8)	0.96	0.09 (0.33–2.81)	0.95	0.09 (0.01–54.2)	0.96
HPV status						
Positive	1		1		1	
Negative	2.98 (1.24–7.22)	0.01	5.12 (1.87–13.9)	0.002	3.65 (1.83–7.26)	0.0002
p53						
Negative	1		1		1	
Positive	2.18 (0.88–5.40)	0.09	1.83 (0.75–4.42)	0.18	2.56 (0.35–18.9)	0.35
Combination of HPV status + p53						
Low risk	1		1		1	
Intermediate risk	0.72 (0.18–2.90)	0.65	0.79 (0.19–3.34)	0.75	1.07 (0.42–2.72)	0.88
High risk	1.81 (1.11–2.97)	0.01	2.01 (1.19–3.36)	0.008	1.75 (1.20–2.57)	0.004

Low risk represents the HPV positive tumors and negative for p53, Intermediate risk represents either HPV positive and positive for p53 or HPV negative and negative for p53, and High risk represents the HPV negative tumors and positive for p53. NA: not available.

**Table 4:** Multivariate analysis for cancer-specific survival, disease-free survival and overall survival of patients with oropharyngeal squamous cell carcinoma.

	Cancer-Specific Survival		Disease-Free Survival		Overall Survival	
	HR (95% CI)	*p*-Value	HR (95% CI)	*p*-Value	HR (95% CI)	*p*-Value
Model 1						
Smoking	4.65 (1.07–20.1)	0.04	7.94 (1.04–60.2)	0.05		
Location	2.30 (1.10–4.78)	0.03	2.23 (1.08–4.63)	0.03	3.12 (1.07–9.60)	0.04
HPV status	3.47 (1.16–10.4)	0.02	3.73 (1.29–10.7)	0.01		
Model 2						
Smoking	5.19 (1.19–22.4)	0.02	9.19 (1.22–69.1)	0.03	7.41 (1.75–31.3)	0.006
Location	2.39 (1.20–4.75)	0.01	2.40 (1.17–4.91)	0.01	2.03 (1.10–3.73)	0.02
Combination of HPV status + p53	2.26 (1.43–3.59)	0.0005	1.88 (1.04–3.41)	0.03	2.30 (1.01–5.24)	0.04

## Data Availability

De-identified clinicopathologic data may be available from the corresponding author upon reasonable request and in accordance with institutional and ethical regulations.
